# A cephalometric method to diagnosis the craniovertebral junction abnormalities in osteogenesis imperfecta patients

**DOI:** 10.4317/jced.52126

**Published:** 2015-02-01

**Authors:** Mercedes Ríos-Rodenas, Joaquín de Nova, María-Pilar Gutiérrez-Díez, Gonzalo Feijóo, Maria-Rosa Mourelle, Mario Garcilazo, Ricardo Ortega-Aranegui

**Affiliations:** 1 Profesora asociada de Odontopediatría (Universidad Alfonso X El Sabio). Investigadora Proyecto. Proyecto (FMM2013AP123942013) Fundación Mutua Madrileña (X Convocatoria); 2 Profesor Titular de Odontopediatría (Universidad Complutense de Madrid (UCM)). Responsable Proyecto. Proyecto (FMM2013AP123942013) Fundación Mutua Madrileña (X Convocatoria); 3 Especialista en Endocrinología Pediátrica. Unidad de Osteogénesis Imperfecta (Hospital Universitario de Getafe (Madrid)). Investigadora Proyecto. Proyecto (FMM2013AP123942013) Fundación Mutua Madrileña (X Convocatoria); 4 Investigador Proyecto. Proyecto (FMM2013AP123942013) Fundación Mutua Madrileña (X Convocatoria); 5 Profesora Contratada Doctora de la UCM. Investigadora Proyecto. Proyecto (FMM2013AP123942013) Fundación Mutua Madrileña (X Convocatoria); 6 Profesor asociado (UCM). Investigador Proyecto. Proyecto (FMM2013AP123942013) Fundación Mutua Madrileña (X Convocatoria)

## Abstract

Osteogenesis imperfecta (OI) is a hereditary bone fragility disorder that in most patients is caused by mutations affecting collagen type I. Their typical oral and craneofacial characteristics (Dentinogenesis imperfecta type I and class III malocclusion), involve the dentist in the multidisciplinary team that treat these patients. It is usual to perform lateral skull radiographs for the orthodontic diagnosis. In addition, this radiograph is useful to analyse the junctional area between skull base and spine, that could be damaged in OI. Pathology in the craneovertebral junction (CVJ) is a serious complication of OI with a prevalence ranging from rare to 37%. To diagnosis early skull base anomalies in these patients, previously the neurological symptoms have been appear, we make a simple cephalometric analysis of the CVJ. This method has four measurements and one angle. Once we calculate the values of the OI patient, we compare the result with the mean and the standard deviations of an age-appropriate average in healthy controls. If the patient has a result more than 2,5 SDs above the age-appropriate average in healthy controls, we should to refer the patient to his/her pediatrician or neurologist. These doctors have to consider acquiring another diagnostic images to be used to determine cranial base measurements with more reliability. Thereby, dentists who treat these patients, must be aware of the normal radiological anatomy of the cervical spine on the lateral cephalogram.

** Key words:**Osteogenesis imperfecta, craniovertebral junction, cephalometric.

## Introduction

Osteogenesis imperfecta (OI), also referred to as “brittle bone disease”, is a genetic disorder of connective tissue that causes increased bone fragility and low bone mass. This disorder is relatively rare with a composite incidence of approximately 6.5 per 100,000 live births and a population prevalence of 1 in 30,000 (1,2).

The most frequently used classification of OI is by Sillence *et al.* in 1979 (3). It outlines four types of OI based on clinical, radiographic, and genetic criteria (1,2). Osteogenesis imperfecta type I includes patients with mild disease and absence of major bone deformities. Type II is lethal in the perinatal period (4). OI type III is the most severe form in children surviving the neonatal pe-riod. These patients are of very short stature and have limb and spine deformities secondary to multiple fractures, which can lead to respiratory difficulties. Patients with mild to moderate bone deformities and variable short stature are classified as osteogenesis imperfecta type IV (1).

This classification has been expanded to include new distinctive types, the most well known types being types V-XI (2). The discovery of new implied genes increase the classification from type XII to type XV (Online Mendelian Inheritance in Man), but most of the cases can be classified into types I–IV OI. It is very possible that the current debate over the classification could bring future changes (5).

Typical oral and craneofacial characteristics, involve the dentist in the multidisciplinary team that treat this patients. OI has several dental and serious oclusal problems. Dentinogenesis imperfecta (DI) Type I is the most common oral problem in OI patients. In addition, dental malocclusions are marked in many OI subjects and include a high incidence of Class III oclusal relationship (70-80% in types III and IV OI), anterior and/or posterior crossbite, and posterior open bite (6-9).

For this reason, it is normal to perform lateral skull radiographs for the orthodontic diagnosis of these patients. With this diagnosis image, the dentist should analyse the junctional area between skull base and spine, that could be damaged in OI.

Craniovertebral junction (CVJ) abnormalities or Cranial base abnormalities are one of the most important complication of OI. It is most commonly seen in type III and IV, with a prevalence ranging from rare to 37%. This may be due to the fact that craniospinal deformity in OI is often asymptomatic (10-13).

Some studies have made a connection between greater prevalence and fenotipic discoveries like that of DI (14,15).

The reason for the development of skull base abnormalities in OI is not known; however, it is stated that the deformation of the skull is due to softness of the skull or to repetitive microfractures in the region of the foramen Magnum. As a result, an infolding of the occipital condyles occurs which is accompanied by an upward migration of the cervical spinal column into the foramen Magnum. Consequently, it could later be associated with a compression of the brainstem and spinal cord that can be asymptomatic or can lead to a variety of neurological symptoms (10-12,16-19).

The current treatments (biphosphonates) could change the personal profile, because one might assume that these treatments might have a preventive effect (11,13,20).

The three most frequent clinical features of these patients were nystagmus, headaches, and ataxia facial numbness. These features are often progressive and can lead to rapid neurological deterioration, respiratory arrest, or even death (11). To prevent these catastrophic symptoms, a neurosurgical treatment could be necessary (21-25).

The most common ages for presentation of skull base abnormalities is between the ages of 11 and 15 years old (18); however, there are reports with a skull base anomalies diagnosis in younger patients (15,18,24,26). For this reason it is important to study the CVJ abnormalities in children with OI.

In addition to the clinical signs and symptoms, numerous imaging diagnosis methods have been suggested for the diagnosis or confirmation of pathology in the basilar region. Although the actual diagnosis is nowadays most accurately carried out from computed tomography (CT) or magnetic resonance imaging (MRI); lateral skull radiographs are still recommended for screening purposes as a simple, low cost, and low radiation method for patients at risk (17,27-30).

Currently we know that the anatomical landmarks for analysis of basilar abnormalities are similarly located on lateral skull radiographs and midsagittal CT or MRI. Therefore, the value of the findings regarding anatomical relationships in the craniovertebral junction does not depend on the imaging modality (15).

To detect skull base anomalies, we measure the distance of the odontoid process to reference lines and the anterior cranial base angle in lateral skull radiographs. But the early diagnosis has been difficult because of the data on normal dimensions of the CVJ and their growth related changes in unaffected children has been limited (27,31).

Arponen et al. (31) analysed longitudinally changes in the vertical dimensions of the CVJ and in the flexion of the anterior skull base in normal growing individuals. Their findings indicate that in 5-6 year old children, the skull base measurements are significantly different from older age groups. Furthermore, studies on patients younger than 9 should at least be provided with age-appropriate controls. Nevertheless, a notable deviation from the documented normal values is suggestive of pathological development in the CVJ.

Dentists, in addition to paying attention to dental complications (32), should analyse the junctional area between the skull base and spine in a lateral skull radiograph of at-risk patients, such as those with OI. This area may reveal a pathologic disorder in asymptomatic subjects, and the dentist may be the first person to detect some of these abnormalities. Progressively degenerative defects, if discerned early, may help in mitigation of the severity of their consequences (33).

## Material and Methods

To decide if an OI patient is going to require an accurate monitoring of their development, we make a simple cephalometric analysis of the CVJ.

-Diagnostic criteria

We used the definitions and diagnostic criteria established by Kovero *et al.* (15) to classify the skull base abnormalities in these patients. Currently these anomalies could be arrange into three groups:

a. Basilar invagination: protrusion of the odontoid process into the foramen Magnum.

b. Basilar impression: position of the odontoid process far above the caudal borders of the skull, without penetrating in the interior of the foramen Magnum.

c. Platybasia: a flat anterior cranial base angle.

-Cephalometric analyses of the CVJ

At first, the cranial and anatomical vertebral points are determined. These are further described in  Table 1.

**Table 1:**
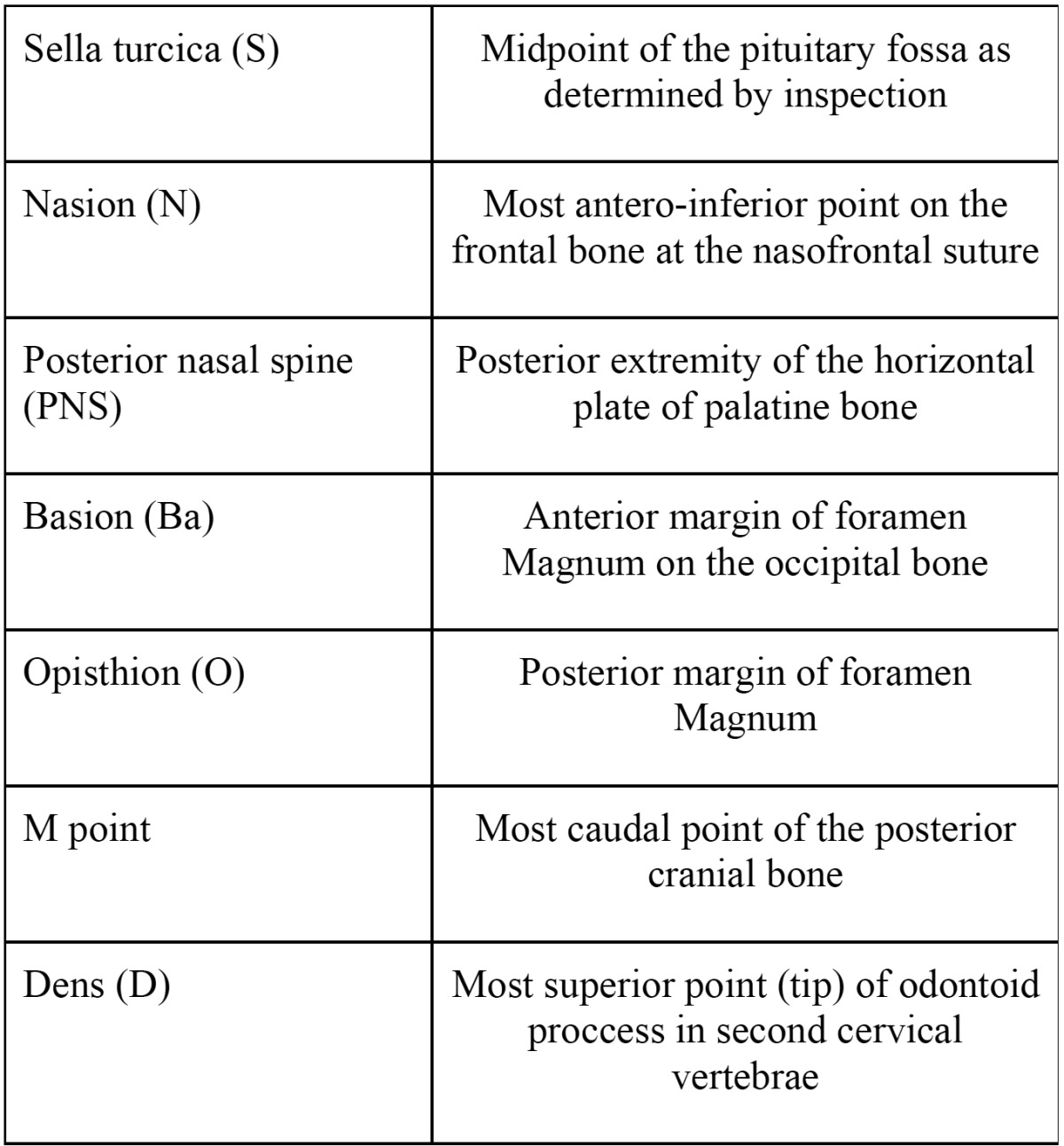
Cephalometric landmarks used in this study.

**Figure 1:**
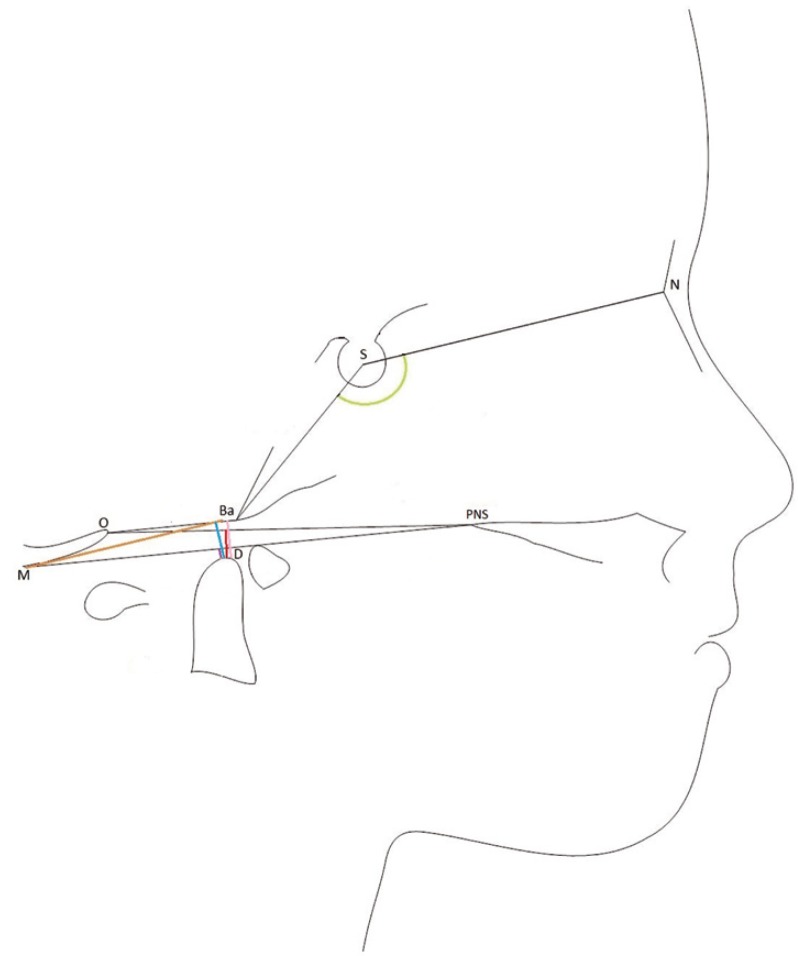
Schematic illustrating the linear and angular variables analysed in the lateral radiographs. N, Nasion; S, Sella; PNS, Posterior Nasal Spine; Ba, Basion; O, Opisthion; D, Dens; M, M point. McRae measure (pink), Chamberlain measure (red), McGregor measure (purple), Kovero measure (blue), anterior cranial base angle (green).

We used a radiographic criteria for each one of the abnormalities. There are four measurements and one angle. We can observe in figure 1 a schematic representation. Any distance had a positive value when the odontoid process lay above the respective line used for each measure, and negative when it lay below (15,20). The radiographic magnification was corrected to measure the linear distances. When magnification was unknown, we used only angular measure (13).

a. To diagnose Basilar invagination, we consider the -McRae measure:

McRae measure: represents the perpendicular distance from the tip of the odontoid process (D) to the McRae line. McRae line or foramen Magnum line joining the anterior (Basion (Ba)) and posterior (Ophistion (O)) margins of the foramen Magnum (34). Abnormal results, defined as a result above 0 for the McRae measure.

b. To diagnose Basilar impression, we consider these three measurements:

-Chamberlain measure: represents the perpendicular distance of the tip of the odontoid process (D) to the Chamberlain line. This line runs from the posterior nasal spine (PNS) to the posterior lip of the foramen Magnum (Ophistion (O)) (35).

-McGregor measure: represents the perpendicular distance of the tip of the odontoid process (D) to the McGregor line. The line joining the posterior nasal spine (PNS) to the most caudal portion of the posterior cranial base (M) (36).

-Kovero measure or DM distance represents the perpendicular distance of the tip of the odontoid process (D) from a parallel line to the Nasion-Sella line passing through the most caudal part of the posterior cranial bone (M) (15).

The radiographic criteria for basilar impressions are fulfilled if the Chamberlain measure, the McGregor measure, or the Kovero measure are elevated by more than 2,5 standard deviations (+2,5 SDs) above the average of age-matched healthy controls (13).

c. To diagnose Platybasia, we consider the anterior cranial base angle:

-Anterior cranial base angle or basal angle is delimited for the Nasion-Sella-Basion landmarks (36). Platybasia is diagnosed when the anterior cranial base angle was more than 2,5 SDs above the average of healthy controls (13).

A lower threshold limit (+2,5SDs) ensures better sensitivity of the screening, whereas a higher limit (+3SDs) yields better specificity of the diagnosis. In an adult population, the emphasis may be on limitation of false-positive findings, whereas in children it is beneficial to identify all subjects that need closer follow-up (13).

Patients who had at least one of these diagnoses were said to have a skull base abnormality (20).

Once we calculate the values of the measurements, we compare the result with the mean and the SDs of a healthy subject. We can find these values on a statistical table devised by Arponen *et al.* They classify the results in males and females and in age groups of a 3 year range. A notable deviation (+2,5 SDs) of our patient, would suggest a pathological development be the craneocervical union (31).

If the patient has a result more than 2,5 SDs above the age-appropriate average in healthy controls, we should to refer the patient to his/her pediatrician or neurologist. These doctors have to consider acquiring another diagnostic images to be used to determine cranial base measurements with more reliability (20).

## Results and Discussion

-Cranial Base Structure in Healthy Young Populations

Although the normal dimension values of the skull base structure and their growth-related changes in healthy children, are a pre-requisite for an accurate diagnosis and a better understanding of the development of CVJ abnormalities, this data has been limited (27,31).

In the past, the diagnostic criteria and the reference values to be analysed in the CVJ in children were extrapolated from reports of adult populations.

Arponen *et al.*, in 2010, analysed longitudinally changes in the vertical dimensions of the CVJ and in the flexion of the anterior skull base in normal growing individuals. They provided age-appropriate normative values (from 3 to 25 years old) for anterior skull base angle, as well as measurements of McGregor’s line, Chamberlain’s line and Kovero’s line.

They showed that in young children, the odontoid process is situated relatively caudally in relation to the skull base structures and reaches a mean level comparable to that of the adults approximately at the age of 7 years in both males and females (31).

Cheung *et al.* analysed the skull base anatomy in healthy populations as well. In the 191 healthy controls that they studied, they found significant statistical differences in age groups in the McRae, Chamberlain, McGregor and Kovero´s measurements. However, after the age of 9 years old, these measurements remained constant. Cranial base angle did not vary significantly with age (20).

The need for age-specific controls for young children was based on earlier observations that most of the skull base measures vary with age during childhood but remain constant in healthy subjects aged 9 years and older (20).

-Skull base abnormalities in OI patients

Even though we know that there is elevated risk of CVJ abnormalities and that their consequences could be death, there are few studies that have analysed these facts in OI patients. One of the reasons for this could be that OI is a rare disorder, and it is difficult to find enough patients for an investigative study.

In addition, there is not consensus regarding diagnostic criteria to classify if an OI patient could present pathology or not.

When reading scientific literature about skull base abnormalities, we found that in the past it was generally considered abnormal if the tip of the odontoid projects more than 5 mm above Chamberlain’s line or 7 mm above McGregor’s basal line. With these diagnostic criteria, Jensen *et al.* reported that the 19% of their OI patients had basilar invagination (12). However, Sawin *et al.* used diagnostic criteria less exhaustive. They consider it a disorder if the odontoid process projects above 0 for the McRae´s line, or more than 2,5 or more tan 4,5 mm above Chamberlain´s or McGregor´s line, respectively. All their 18 OI patients had basilar invagination, because it was inclusive criteria (37).

Engelbert *et al.* published cranial base pathology in 17% of their 47 OI patients (from 1 to 16 years old). They used the McGregor´s measure, but did not explain the limits to established pathology (14).

Janus and colleagues, in 2003, suspected basilar invagination in 10% of their 130 pediatric OI patients based on ‘‘protrusion of the odontoid above Chamberlain’s or McGregor’s line’’ on lateral skull radiographs (10,20).

Kovero *et al.* evaluated with a cephalometric analysis of the skull base, 54 OI patients and 108 control volunteers. They proposed new diagnostic limits to be used in the adult population, independent of the imaging modality (15).

Cheung *et al.* reported that 22% of patients were positive for at least one skull base abnormality. Platybasia was by far the most prevalent diagnosis, affecting 16% of patients, whereas basilar impression and basilar invagination were noted in 6% and 4% of patients, respectively (20).

Arponen *et al.* observed in 2012 CVJ anomalies in 37% of their patients OI studied. Of the three types of anomalies, basilar invagination was seen in 13%, basilar impression in 15%, and platybasia in 29% of the patients. At a group level, they found no evidence of progressive CVJ pathology with age. A higher risk of having any of the pathological conditions was associated with a lower height, so they suggested a careful follow-up of cranial base anomalies particularly in subjects with OI and severe growth failure (13).

The skull base abnormalities may emerge in skeletal disorders already in infancy. An initial evaluation with a lateral skull radiograph can provide useful information about them in patients with OI (20). It should be carried out for all patients before school age. In the case of normal findings from the image(s) taken, further imaging is unnecessary in symptomless patients to keep the patient radiation dose at a minimum. In case of abnormal findings in a radiograph or MR image, an individually adjusted plan for follow-up and treatment is warranted (13).

Dentists must be aware of the normal radiological anatomy of the cervical spine on the lateral cephalogram. Many abnormalities of the cervical spine do not manifest themselves symptomatically until adolescence or young adulthood.
